# 45 years of tetracycline post exposure prophylaxis for STIs and the risk of tetracycline resistance: a systematic review and meta-analysis

**DOI:** 10.1186/s12879-024-09275-3

**Published:** 2024-04-04

**Authors:** Thibaut Vanbaelen, Sheeba Santhini Manoharan-Basil, Chris Kenyon

**Affiliations:** 1 STI Unit, Department of Clinical Sciences, Institute of Tropical Medicine, 2000 Antwerp, Belgium; 2 Division of Infectious Diseases and HIV Medicine, University of Cape Town, Cape Town, 7700 South Africa

**Keywords:** *Doxycycline PEP*, Tetracycline, Minocycline, AMR, PrEP, Gonorrhoea

## Abstract

There is considerable interest in the use of doxycycline post exposure prophylaxis (PEP) to reduce the incidence of bacterial sexually transmitted infections (STIs). An important concern is that this could select for tetracycline resistance in these STIs and other species. We searched PubMed and Google Scholar, (1948–2023) for randomized controlled trials comparing tetracycline PEP with non-tetracycline controls. The primary outcome was antimicrobial resistance (AMR) to tetracyclines in all bacterial species with available data. Our search yielded 140 studies, of which three met the inclusion criteria. Tetracycline PEP was associated with an increasedprevalence of tetracycline resistance in *Neisseria gonorrhoeae*, but this effect was not statistically significant (Pooled OR 2.3, 95% CI 0.9-3.4). PEP had a marked effect on the *N. gonorrhoeae* tetracycline MIC distribution in the one study where this was assessed. Prophylactic efficacy was 100% at low MICs and 0% at high MICs. In the one study where this was assessed, PEP resulted in a significant increase in tetracycline resistance in commensal *Neisseria* species compared to the control group (OR 2.9, 95% CI 1.5-5.5) but no significant effect on the prevalence of tetracycline resistance in *Staphylococcus aureus*. The available evidence suggests that PEP with tetracyclines could be associated with selecting tetracycline resistance in *N. gonorrhoeae* and commensal *Neisseria* species.

**Supplementary Information:**

The online version contains supplementary material available at 10.1186/s12879-024-09275-3.

## Introduction

The incidence of bacterial sexually transmitted infections (STIs), such as *Neisseria gonorrhoeae* (*Ng*) infections, has been increasing globally for the last decades [1]. This increase has led to the search for novel interventions to reduce STI incidence [2]. One of these interventions, the use of a tetracycline, typically doxycycline, after unprotected sex, has gained increased attention in recent years [3]. Four randomized controlled trials (RCTs) have found that this doxycycline post exposure prophylaxis (PEP) can reduce the incidence of chlamydia and syphilis in men who have sex with men (MSM) [3–6]. Three of these trials found a reduced incidence of gonorrhoea. The median doxycycline consumption in these trials ranged from 8 to 30 defined daily doses (DDD) per month, which raised concerns that doxycycline PEP could induce tetracycline resistance in a range of bacterial species, including in *Ng* [3, 5, 7]. This is of particular concern, given that *Ng* has evolved resistance to all the classes of antimicrobials used to treat it and might become untreatable in the near future [8, 9]. In the case of tetracyclines, low level resistance is conferred by chromosomal mutations in *rpsJ*, *porB* and *mtrR* [8]. High level resistance is typically due to the acquisition of the *tetM* gene on a plasmid [8]. The spread of both types of resistance is predominantly clonal in *Ng* [10].

Only two of these RCTs have evaluated the effect of doxycycline on tetracycline resistance in *Ng* but the duration of follow-up was short, and the number of isolates tested were small [3, 5]. In addition, one of these RCTs evaluated the effect of doxycycline PEP on the prevalence of doxycycline resistance in *Staphylococcus aureus* and commensal *Neisseria* species [5, 11]. The authors of these studies have concluded that the risk of antimicrobial resistance (AMR) is small or non-existent [3, 5, 11]. These conclusions have, in turn, led to doxycycline PEP being offered to MSM attending certain STI clinics [12].

In contrast, an RCT in women in Africa found that doxycycline PEP had no effect on the incidence of chlamydia, gonorrhoea or syphilis [13]. An older RCT from 1979 in men in the US Navy found that although minocycline PEP reduced the incidence of gonorrhoea by 54% overall, this effect was driven by a reduction in the incidence of gonococcal infections with low minocycline MICs [14]. Minocycline PEP had no effect on the incidence of infections with higher tetracycline MICs. The authors concluded that minocycline PEP would likely select for gonococcal AMR and was thus not advisable.

In this systematic review we summarize the available evidence from RCTs as to the association between tetracycline PEP and tetracycline resistance in all bacterial species with available data. Assessing the impact of tetracycline PEP on resistance is crucial to inform decisions regarding the broader implementation of such practices.

## Materials and methods

This systematic review was not published in a registry and no protocol was prepared. This review was reported according to the PRISMA guidelines [15]. All the steps were performed independently by two reviewers (CK and TV). The PRISMA checklists are presented in STable 1.

### Search strategy

PubMed and Google Scholar were searched for articles and conference abstracts published between 1 March 1948 (first report of tetracyclines in the scientific literature) and 30 April 2023. Reference lists of relevant articles were checked for additional titles for inclusion in the review. Keywords used for the search included “postexposure prophylaxis”, “tetracycline”, “doxycycline”, “minocycline”, “sexually”, and “gonorrhoea” (STable 2).

### Selection process and criteria

The titles and abstracts of all the articles were screened by two independent reviewers (CK and TV). Duplicates were then manually removed.

Studies were included or excluded according to the following predefined criteria:

### Inclusion criteria

1. Randomized controlled trial study design.
2. Compare the efficacy of tetracycline with either placebo or no treatment for reducing the incidence of bacterial STIs (syphilis/gonorrhoea/chlamydia).
3. Abstracts and full text available.
4. Report the prevalence of tetracycline resistance in any bacterial species at baseline and study end.

There were no restrictions on the language that the study was published.

### Data extraction and synthesis

Data on study characteristics and outcomes were independently extracted by two review authors (CK and TV) into the Review Manager software (RevMan, version 5.4.1, Cochrane, London, UK). Discrepancies were resolved by discussion with the third author (SB). The authors of the Luetkemeyer and Molina studies were contacted to provide the individual MIC data of isolates from their studies [3, 5].

Two types of resistance data were extracted:

1. Tetracycline MIC distribution of isolates. This data was only available for *N. gonorrhoeae* from a single study (Harrison et al.). The tetracycline MIC distribution of gonococcal isolates per species of the two arms for the tetracycline and placebo groups post PEP were compared with the Mann-Whitney test. The tetracycline MIC distribution of isolates per species of the two arms post PEP were compared with the Mann-Whitney test.
2. The proportion of isolates resistant to tetracycline. This variable was calculated for each bacterial species as the number of individuals with tetracycline resistant isolates cultured per total number of individuals with this same species cultured. If only MIC distribution was available, we calculated the proportion of tetracycline resistance using the resistance thresholds used in the other studies included this review, in order to allow for comparison (≥ 1 or 2 mg/L). The number of events and the number of participants included in the control and intervention groups of each study were extracted. Data was extracted for all bacterial species with available data.

Data was also extracted on the year and country in which the study took place, the target bacterial species assessed for tetracycline susceptibility, the sampling and study methodology, the tetracycline PEP protocol used, the method used to assess MICs and the study primary outcome.

The Odds ratio (OR) and the 95% confidence intervals were calculated for the proportion of isolates resistant to tetracycline. Heterogeneity was assessed via the I^2^ statistics and the p- value of the chi-square statistics. A fixed-effects model was used to combine the results for the meta-analysis. Publication bias was not assessed as only three studies were assessed. All analyses were conducted in Review Manager software (RevMan, version 5.4.1, Cochrane, London, UK) or STATA v16.1. When necessary, data from figures were digitized using WebPlotDigitizer (https://apps.automeris.io/wpd/). A *P*-value of < 0.05 was considered statistically significant.

### Risk of bias assessment

The risk of bias was assessed by two independent reviewers (CK and TV) using the Cochrane risk-of-bias tool for randomised trials version 2 (RoB 2). As prescribed by the RoB 2 tool, each study was assessed in six domains: (1) randomisation process, (2) allocation concealment, (3) blinding of participants and personnel, (4) blinding of outcome assessment, (5) incomplete outcome data, and (6) selective reporting. Each domain was assessed as ‘low risk of bias’, ‘some concerns’ or ‘high risk of bias’. The assessment for domains 5 and 6 was based on outcome data for culture and tetracycline susceptibility testing of *N. gonorrhoeae* since this was an outcome of each included study and a primary objective of our analysis. Assessment of each of the 6 domains led to an overall risk of bias assessment. Any disagreements were resolved by discussion between the two reviewers.

## Results

### Study characteristics

**Fig. 1:**
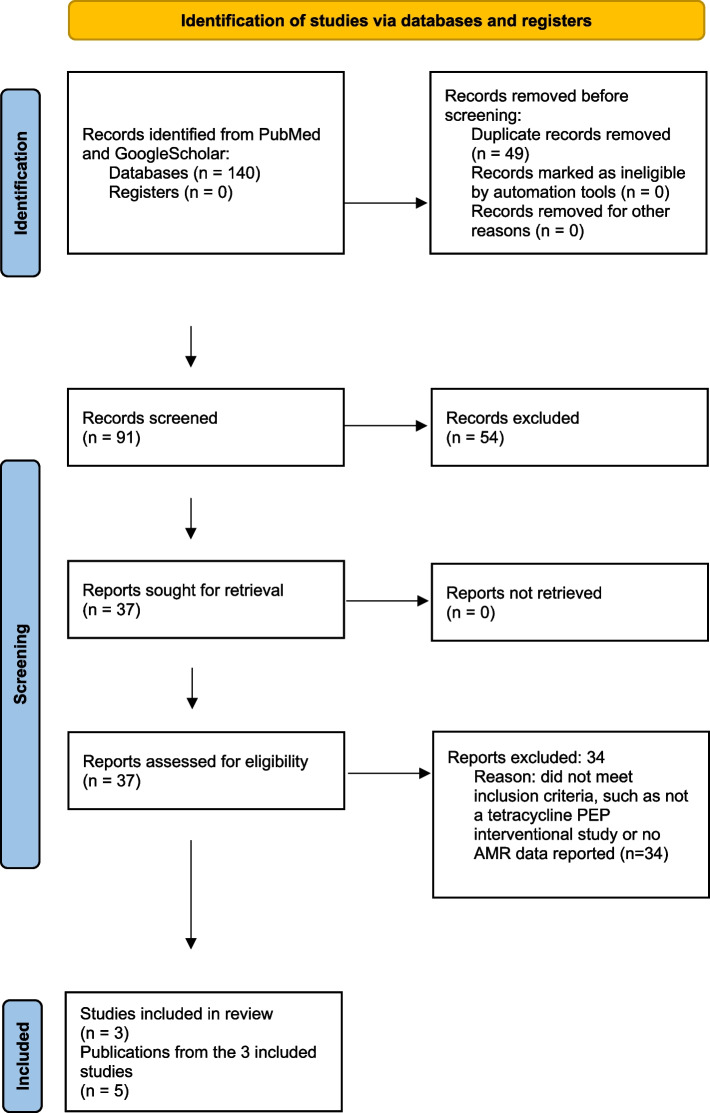
PRISMA flowchart of study selection

The literature search identified 140 studies (Fig. 1). Of these, 49 were excluded due to duplication, 54 were excluded based on title and abstract, and 37 full-text articles were reviewed. Five of these articles met the inclusion criteria and were included in the review (Table 1). These five articles described the findings of three RCTs. All three of these RCTs reported tetracycline resistance in *N. gonorrhoeae,* one in commensal *Neisseria* spp., one in *Mycoplasma genitalium,* and one in *Staphylococcus aureus.* In one of these RCTs, the authors only reported the MIC distribution of individual isolates, for the two remaining RCTs only the proportion of resistant isolates were reported, without individual MICs (Table 1).

**Table 1:** Selected characteristics of the studies included in the systematic review

Study, Year of publication, references	Target bacterial species assessed for tetracycline susceptibility	Country	Study Population/method	PEP protocol	MIC testing methodology	Primary outcome
Harrison 1979 [14]	*N. gonorrhoeae*	USA	1080 men from a US Navy ship after sexual exposure during 2 periods of leave in a port in East Asia randomized to either 200 mg minocycline or placebo after each sexual exposure, but maximum 200 mg per 18 hour period. 815 of these men were reinterviewed and had urethral samples taken at a single time point. MIC results available for 62 isolates of *N. gonorrhoeae* out of 81 incident *N. gonorrhoeae* infections.	200 mg minocycline after each sexual exposure (median time between sex and PEP 8 hours, range 30 min-36 hours)	Agar dilution with tetracycline. MICs of each isolate reported. Threshold for definition of tetracycline resistance not defined	Overall, 54% reduction in culture positive urethral gonorrhoea. No effect on infections with elevated minocycline MIC
Molina 2018 [3, 16]	*N. gonorrhoeae, M. genitalium*	France	232 MSM randomized 1:1 to doxycycline (200 mg) or no prophylaxis within 24 hours post each episode of condomless sex. Follow up was 10 months. NG tested for via quarterly NG PCR of the pharynx/rectum/urine. The primary analysis was mITT. The primary outcome was the occurrence of a first STI, defined as first episode of syphilis, chlamydia, or gonorrhoea infection. MIC results were provided for 9 isolates of *N. gonorrhoeae* out of 57 incident *N. gonorrhoeae* infections. Culture was only attempted in 28 of the infections.	200 mg doxycycline within 24 hours after condomless sex	Agar dilution with tetracycline. Resistance defined as ≥1 mg/L. Only proportion of resistant isolates were reported (MICs of each isolate were not reported)	Occurrence of a first STI was lower in the doxyPEP arm than in the placebo arm (HR 0.53). Doxycycline reduced the occurrence of a first episode of chlamydia (HR 0.3) and syphilis (HR 0.27). The effect on gonorrhoea was not significant.
Luetkemeyer 2023 [5, 11]	*N. gonorrhoeae, commensal Neisseria species, S. aureus*	USA	501 MSM and transgender women (327 in the PrEP cohort and 174 in the PLWH cohort), with a history of chlamydia, gonorrhoea or syphilis in the past 12 months were randomized 2:1 to doxycycline (200 mg) or placebo within 72 hours post each episode of condomless sex. Followed up quarterly for 12 months. NG tested for via quarterly NG PCR of the pharynx/rectum/urine. The primary analysis was mITT. The primary outcome was the incidence of at least one bacterial STI (gonorrhea, chlamydia, syphilis) each quarter. Tetracycline susceptibility data available for 29 isolates of *N. gonorrhoeae* out of 157 incident *N. gonorrhoeae* infections, for 162 isolates of *S. aureus* and for 178 isolates of commensal *Neisseria* spp.	200 mg doxycycline within 72 hours after condomless sex	Agar dilution with tetracycline. Resistance defined as ≥2 mg/L. Only proportion of resistant isolates were reported (MICs of each isolate were not reported)	Doxycycline reduced the incidence of at least one diagnosed STI (RR 0.34 in PrEP cohort and 0.38 PLWHIV), of gonorrhoea (RR 4.5/0.43 respectively), chlamydia (RR 0.12/0.26, respectively) and syphilis (0.13/0.23, respectively)

*Abbreviations: mITT* Modified intention to treat, *MSM* men who have sex with men, *NG Neisseria gonorrhoeae*, *PLWH* People living with HIV, *PrEP* preexposure prophylaxis, *RR* relative risk, *HR* hazard ratio

In the Harrison study, 1080 sailors were randomized to either 200 mg minocycline or placebo after sex with sex workers during two episiodes of shore leave. Eight hundred fifteen of these men were reinterviewed and had urethral samples taken at a single time point. The MICs of *N. gonorrhoeae* infections were assessed with agar dilution, and MIC results were available for 62 isolates of *N. gonorrhoeae* out of 81 incident *N. gonorrhoeae* infections.

The Molina study randomized 232 MSM 1:1 to doxycycline (200 mg) or no prophylaxis within 24 hours post each episode of condomless sex. Follow-up was for 10 months. *N. gonorrhoeae* was tested quarterly via PCR of the pharynx/rectum/urine. The gonococcal MICs were assessed via agar dilution, but only the proportion resistant were reported. Resistant results were provided for 9 isolates of *N. gonorrhoeae* out of 57 incident *N. gonorrhoeae* infections. Culture was only attempted in 28 of the infections.

In the Luetkemeyer study, 501 MSM were randomized 2:1 to doxycycline (200 mg) PEP or placebo. They were followed up quarterly for 12 months. *N. gonorrhoeae* was tested via quarterly PCR of the pharynx/rectum/urine. Tetracycline susceptibility data (tested for via agar dilution, but reported as susceptible or resistant) was available for 29 isolates of *N. gonorrhoeae* out of 157 incident *N. gonorrhoeae* infections, for 162 isolates of *S. aureus* and for 178 isolates of commensal *Neisseria* spp.

### Assessment of risk of bias

**Fig. 2:**
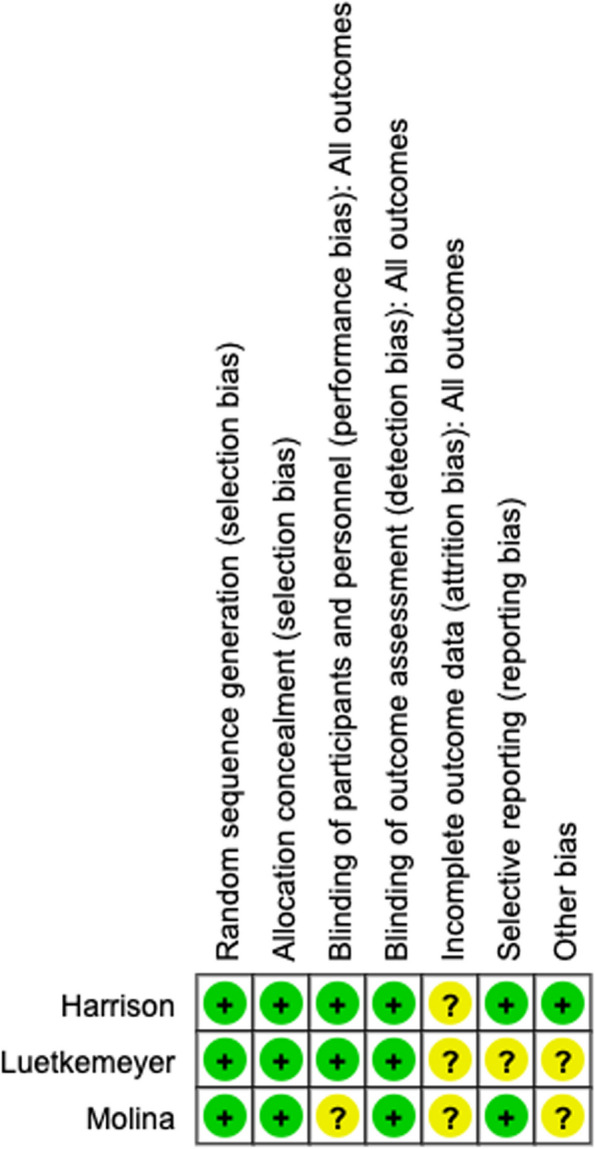
Risk of bias assessment. Key:  + indicates a low risk of bias, ? indicates an unclear risk of bias

The Molina study was an open label study and thus at high risk for bias for blinding of participants and staff (Fig. 2). Both the Molina and Luetkemeyer studies had low culture positivity rates for *N. gonorrhoeae.* In the Molina study, for example, of the 57 incident gonococcal infections detected, culture was only attempted in 28 and was only successful in 9 infections. Because the Molina study was not blinded, it is possible that there was a selection bias in which infections were subjected to culture. The Luetkemeyer study had considerable attrition of samples at the later study visits (Fig. 2; Table 1). The Molina and Luetkemeyer studies were longitudinal studies of cohorts followed up for 10 to 12 months from defined geographical areas. Participants from one arm could have sex with participants from the other arm or partners of these individuals. This could lead to transmission of tetracycline resistant or susceptible *Neisseria* spp. and staphylococci between arms, hence a bias to the null hypothesis [17]. The Harrison study was conducted during two periods of shore leave, and thus, the risk of this contamination between arms was smaller.

### Tetracycline resistance in *N. gonorrhoeae*

- Proportion resistant

**Fig. 3:**
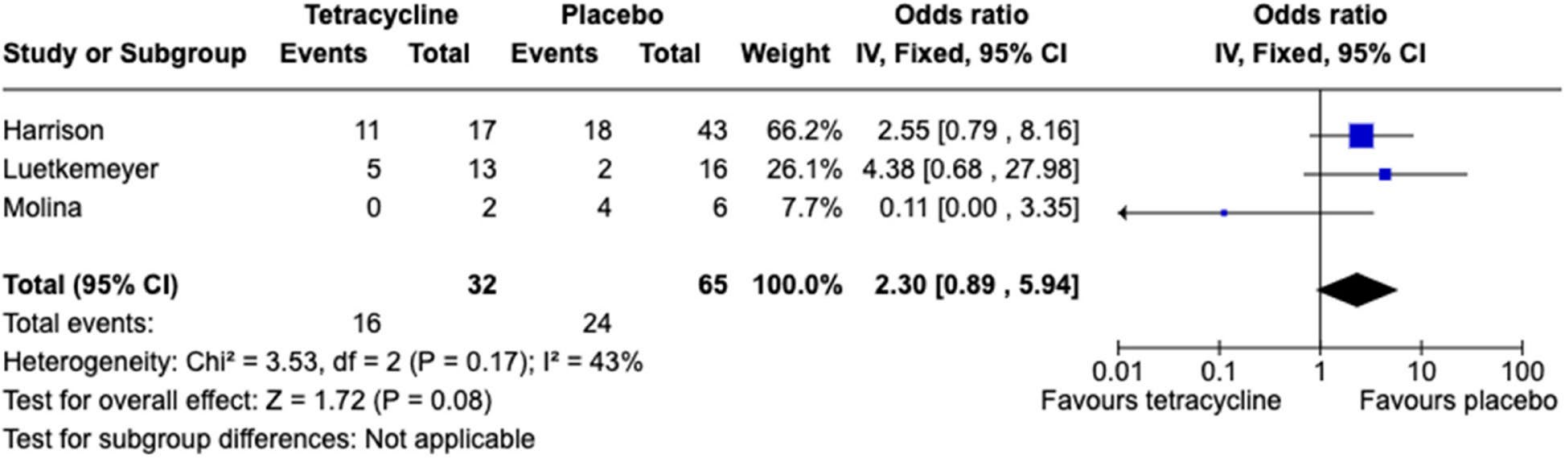
Forest plot showing individual study and pooled ORs (log scale) for tetracycline resistance in *N. gonorrhoeae* and exposure to tetracycline PEP. The tetracycline resistance threshold was ≥1 mg/L for the Molina study and ≥ 2 mg/L for the other two studies

Only two studies included more than 10 isolates of *N. gonorrhoeae*. In these studies, the prevalence of tetracycline resistance was higher in the tetracycline arm – Harrison study OR 2.6 (95%CI 0.8-8.2), Luetkemeyer study OR 4.4 (95%CI 0.7-28.0; Fig. 3). Likewise, the prevalence of tetracycline resistance was higher in the pooled estimates (OR 2.3; 95% CI 0.9-5.9). This pooled estimate was comprised of the proportion resistant at ≥1 mg/L for the Molina study and ≥ 2 mg/L for the other two studies (Fig. 3). The lower bound of the 95% confidence interval crossed one in all these analyses.

b. Effect on MIC distribution

**Fig. 4:**
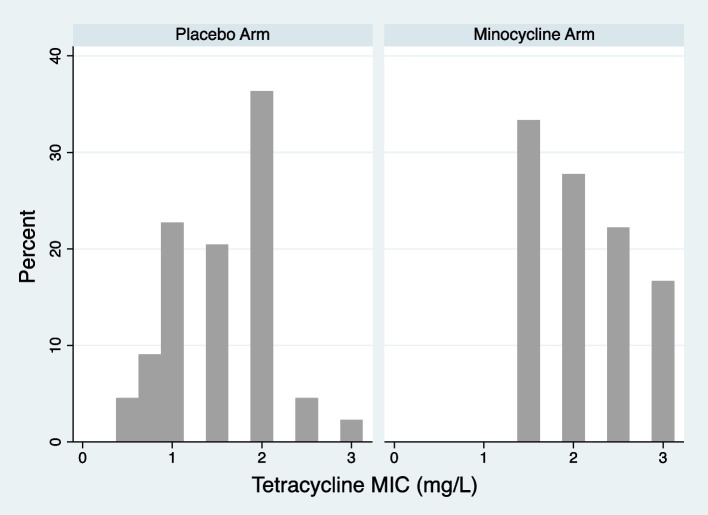
Histograms of the *Neisseria gonorrhoeae* tetracycline MIC distributions in the placebo and minocycline PEP arms of the Harrison et al., study (*P* = 0.0018, Mann Whitney test)

The *N. gonorrhoeae* tetracycline MICs were significantly higher in the minocycline (median 2 mg/L IQR 1.5-2.5 mg/L) than the placebo arm (median 1.5 mg/L IQR 1-2 mg/L; *P* = 0.0018; Fig. 4). None of the gonococcal isolates in the minocycline arm had MICs < 1.5 mg/L whereas 10/44 (22.7%) of the isolates in the placebo arm had MICs in this range (Fig. 4).

### Tetracycline resistance in commensal *Neisseria* species

The prevalence of tetracycline resistance in commensal *Neisseria* species was higher in the tetracycline than the placebo arm in the Luetkemeyer study - the only study where this was assessed (OR 2.9, 95% CI 1.5-5.4).

### Tetracycline resistance in *Staphylococccus aureus*

The prevalence of tetracycline resistance in *S. aureus* was higher in the tetracycline arm in the only study where this was assessed (Luetkemeyer study; OR 2.1; 95% CI 0.4-12.0). The lower bound of the 95% confidence interval crossed one in this analysis.

### Tetracycline resistance in *Mycoplasma genitalium*

The number of samples analyzed for 16 s rRNA mutations at baseline (*n* = 11) and 6 months (*n* = 5) was very small in the only study where this was assessed (Molina study). At 6 months, there was little difference in the prevalence of suspected tetracycline resistance between the tetracycline (1/2) and no tetracycline arms (0/3) [16].

## Discussion

**Fig. 5:**
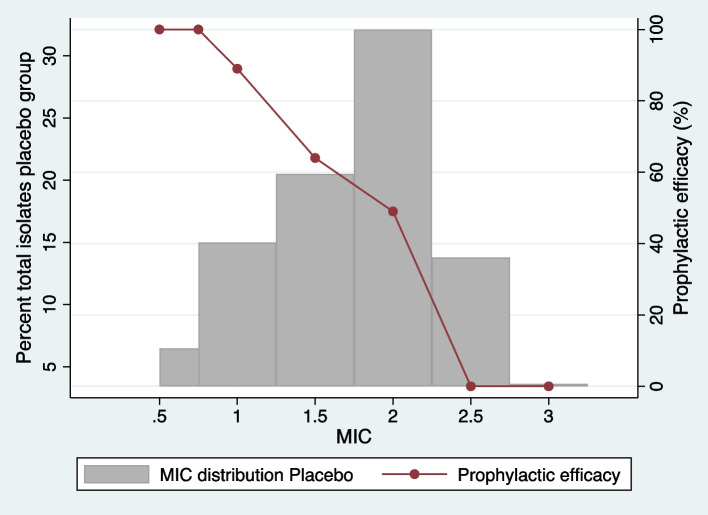
Illustration of the relationship between gonococcal tetracycline MIC and calculated prophylactic efficacy of minocycline in preventing gonorrhoea in the Harrison study. Prophylactic efficacy is defined as the calculated percent of gonorrhoea cases prevented. (Figure produced by the digitalization of two figures in Harrison et al. [14])

Our review found that tetracycline PEP was associated with reduced gonococcal susceptibility to tetracycline When assessed according to the proportion of gonococcal isolates resistant to tetracycline (≥1 or ≥ 2 mg/L), tetracycline PEP was associated with an increased prevalence of tetracycline resistance but this effect was not statistically significant (OR 2.3; 95% CI 0.9-5.9). When MIC distribution was used as the outcome measure, PEP had a more pronounced effect. In the Harrison study, which was the only study where MIC distributions were assessed, the calculated efficacy of PEP varied from 100% if the MICs were < 1 mg to around 50% with intermediate MICs (1 mg to 2 mg) and 0% with MICs > 2 mg/L (Fig. 5). It would be useful to assess if MIC distributions were similarly affected in the other two RCTs. We have requested this MIC distribution data from these RCTs, but the corresponding authors were unable to provide this data in the available time.

An important reason for the difference between these two outcome measures may be that changes in MIC distribution is a more sensitive measure of the impact of an antimicrobial than the proportion of isolates that are resistant where MIC distribution is dichotomized into resistant or susceptible. This process of dichotomization results in a loss of information which, particularly in the setting of small sample sizes, increases the probability of type II errors, and the probability of determining between group differences may be affected by the tetracycline concentration used to define resistance (STable 3) [18, 19]. A number of studies have, therefore, found that testing the MICs of individual colonies is a more sensitive method to detect the effect of antimicrobials on antimicrobial resistance than testing the proportion of colonies with resistance [14, 19]. The Harrison study also found that the incubation period of gonorrhoea was longer in the minocycline group than in the placebo group. Furthermore, in the minocycline (but not the placebo) group, the mean incubation period was longer in isolates with MICs ≤2 mg/L (2.7 days) versus those with MICs > 2 mg/L (5.6 days) [14]. These findings provide further evidence that minocycline was exerting a differential effect according to minocycline susceptibility.

The study with the largest number and proportion of gonococcal isolates assessed for tetracycline susceptibility was the Harrison study. The authors concluded that this differential efficacy would be expected to place significant selection pressure for the emergence of gonococcal resistance to tetracyclines. As a result, they argued against the introduction of minocycline PEP. This conclusion is commensurate with the conclusions derived from other strategies involving the mass use of antimicrobials to reduce the incidence of STIs and related pathogens. A number of placebo controlled trials in the 1940s found that oral penicillin and sulfathiazole PEP reduced the incidence of syphilis, chancroid and gonorrhoea [20, 21]. However, as early as 1949, concerns were raised about the effects on antimicrobial resistance [20]. Likewise, efforts to control gonorrhoea through the mass administration of penicillin in Greenland in the 1950s resulted in a small, temporary reduction in gonococcal prevalence but at the expense of a large increase in penicillin resistance [22]. Mass treatment to reduce meningococcal carriage has similarly been noted to reduce prevalence but at the expense of AMR. As an example, sulfadiazine was used extensively in the US military to prevent meningococcal disease from the 1950s, but this usage was stopped in the 1960s when this programme was implicated in the rapid and extensive emergence of AMR [23]. These findings led researchers to urge caution in the widespread use of antimicrobials to reduce the prevalence of these bacteria [14, 22, 23]. RCTs of doxycycline versus placebo to prevent travellers’ diarrhoea have come to similar conclusions [24–26]. As an example, an RCT found that doxycycline reduced the incidence of travellers’ diarhoea, but at the cost of a higher prevalence of doxycycline resistance – 100% versus 53.3% in the doxycycline and placebo arms, respectively [24]. This plus the risk of microbiome disruption and increased probability of acquiring other multidrug resistant bacteria has led guideline committees to recommend against the routine use of antimicrobials to prevent travellers’ diarrhoea [26, 27].

As far as the commensal *Neisseria* spp., were concerned, the prevalence of tetracycline resistance at the end of the Luetkemeyer study was higher in the doxycycline than in the placebo recipients [11]. This finding was in contrast to the lack of difference found in this study in gonococcal tetracycline resistance. One possible explanation is the larger sample size of commensal *Neisseria* (*n* = 176) compared to *N. gonorrhoeae* (*n* = 27) isolates evaluated. Of note, the odds ratio point estimate for tetracycline resistance between arms for *N. gonorrhoeae* (OR 4.4) was of similar order of magnitude to that for the commensal *Neisseria* spp. (OR 2.9; Table 2, Fig. 3). Other explanations are, however, possible increase in tetracycline resistance between baseline and month 12 in the doxycycline arm was not statistically significant. The difference in tetracycline resistance between arms at month 12 was driven predominantly by a reduced prevalence of tetracycline resistance in the placebo arm at month 12 (STable 4). The sample sizes at month 12 were roughly one-third of the size as the baseline, meaning that the lower prevalence of tetracycline resistance in the placebo arm at month 12 may represent stochastic variations related to small sample sizes. We did not find a significant difference between the Luetkemeyer study arms in the proportion of *S. aureus* with tetracycline resistance at month 12. However, the authors, reported a statistically significant increase in *S. aureus* tetracycline resistance from 5% at baseline to 13% at month 12 in the doxycycline arm only [11].

**Table 2:** Prevalence of tetracycline resistance in *N. gonorrhoeae*, commensal *Neisseria* species and *S. aureus* in the three included studies

Study, Year of publication	Target bacterial species	Tetracycline breakpoint (mg/L)	Baseline		Month 12 (During follow up for *N. gonorrhoeae*)	
			Tetracycline arm N resistant/N tested (% resistant to tetracycline)	Placebo arm N resistant/N tested (% resistant to tetracycline)	Tetracycline arm N resistant/N tested (% resistant to tetracycline)	Placebo arm N resistant/N tested (% resistant to tetracycline)
Harrison 1979 [14]	*N. gonorrhoeae*	≥1 mg/L	NA	NA	18/18 (100%)	38/44 (86.4%)
Harrison 1979 [14]	*N. gonorrhoeae*	≥2 mg/L	NA	NA	12/18 (66.7%)	19/44 (43.2%)
Molina 2018 [3]	*N. gonorrhoeae*	≥1 mg/L	NA	NA	0/2 (0%)	4/6 (66.7%)
Luetkemeyer 2023 [5]	*N. gonorrhoeae*	≥2 mg/L	2/7 (28.6%)	2/8 (25%)	5/13 (38.5%)	2/16 (12.5%)
	Commensal *Neisseria* spp.	≥2 mg/L	189/302 (62.6%)	92/153 (60.1%)	85/122 (69.7%)	25/56 (44.6%)*
	*S. aureus*	≥2 mg/L	12/334 (3.6%)	19/161 (11.8%)	16/137 (11.7%)	3/62 (4.8%)

* *P* < 0.01

Our review only identified three studies for inclusion. For the Molina and Luetkemeyer studies, there was a risk of contamination between arms; the number of isolates of *N. gonorrhoeae* cultured was very low, and these isolates comprised a small proportion of all incident infections. The problems in these two studies were compounded by them not considering the effect on MIC distributions. These considerations mean that we need to be cautious about the conclusions we draw. A number of other limitations are important. The selected studies span 4 decades, were performed in heterogenous populations, used different methods for the diagnosis of *N. gonorrhoeae* (urethral culture versus nucleic acid amplification tests of urine/pharyngeal/rectal samples), and the circulating *N. gonorrhoeae* in the various studies likely had different tetracycline MICs at baseline. In addition to selecting for resistance to tetracyclines, tetracycline PEP could select for resistance to other classes of antimicrobials via bystander selection [10, 28, 29]. This bystander effect could be in STIs or other bacteria. Antimicrobials have also been shown to select for AMR at both individual and population levels [30, 31]. We were unable to evaluate these risks as none of the studies we included in this review evaluated these effects. A different systematic review has, however, noted a number of examples of studies where tetracycline use resulted in reduced susceptibility to tetracyclines in a range of bacterial species [32]. Future studies are required to assess the effects of tetracycline PEP on other important parameters, such as bacterial tolerance to antimicrobials and the human associated microbiome [7]. Studies have estimated that tetracycline PEP could reduce the use of broad-spectrum antimicrobials such as ceftriaxone and azithromycin, but at the cost of an up to 90-fold increase in doxycycline consumption [33, 34]. We were unable to evaluate the net effects of these changes in the antimicrobial consumption in our review. Conversely, doxycycline PEP could select for collateral resistance in other antimicrobials [7]. To our knowledge, the only doxycycline PEP study where this has been assessed, was a substudy of the Molina study which found that doxycycline PEP did not have an effect on the prevalence of macrolide and fluoroquinolone resistance in *Mycoplasma genitalium* [16]. However, this substudy was limited by a very small sample size, as only five *Mycoplasma genitalium* isolates are reported after 6 months of doxycycline PEP. Finally, we may have missed relevant publications due to shortcomings in our search terms and our failure to search trial registries for relevant trials.

We conclude that the available evidence suggests that PEP with tetracyclines such as doxycycline and minocycline is associated with selecting tetracycline resistance in *N. gonorrhoeae* and commensal *Neisseria* species. Because tetracycline resistance spreads clonally and is strongly associated with resistance to other antimicrobials such as ceftriaxone and ciprofloxacin in *N. gonorrhoeae* and other pathogens, the widespread use of doxycycline PEP could select for resistance to not only tetracyclines but also other antimicrobials [10, 29, 35, 36]. In regions where the prevalence of tetracycline resistance is very high, this effect may be less pronounced [7]. Finally, this systematic review should inform future research studies, and should be updated as new evidence emerges.

### Supplementary Information

**Supplementary Material 1.**

## Data Availability

All data generated or analysed during this study are included in this published article.
